# Antioxidant and Antimelanogenic Activities of Kimchi-Derived *Limosilactobacillus fermentum* JNU532 in B16F10 Melanoma Cells

**DOI:** 10.4014/jmb.2104.04008

**Published:** 2021-05-07

**Authors:** Ziyao Meng, Sejong Oh

**Affiliations:** Division of Animal Science, Chonnam National University, Gwangju 61186, Republic of Korea

**Keywords:** Melanin, antioxidant, tyrosinase, *Limosilactobacillus*, microphthalmia-associated transcription factor

## Abstract

Melanin is a natural skin pigment produced by specialized cells called melanocytes via a multistage biochemical pathway known as melanogenesis, involving the oxidation and polymerization of tyrosine. Melanogenesis is initiated upon exposure to ultraviolet (UV) radiation, causing the skin to darken, which protects skin cells from UVB radiation damage. However, the abnormal accumulation of melanin may lead to the development of certain skin diseases, including skin cancer. In this study, the antioxidant and antimelanogenic activities of the cell-free supernatant (CFS) of twenty strains were evaluated. Based on the results of 60% 2,2-diphenyl-1-picrylhydrazyl scavenging activity, 21% 2,2'-azino-bis (3-ethylbenzthiazoline-6-sulfonic acid) scavenging capacity, and a 50% ascorbic acid equivalent ferric reducing antioxidant power value, *Limosilactobacillus fermentum* JNU532 was selected as the strain with the highest antioxidant potential. No cytotoxicity was observed in cells treated with the CFS of *L. fermentum* JNU532. Tyrosinase activity was reduced by 16.7% in CFStreated B16F10 cells (but not in the cell-free system), with >23.2% reduction in melanin content upon treatment with the *L. fermentum* JNU532-derived CFS. The inhibitory effect of the *L. fermentum* JNU532-derived CFS on B16F10 cell melanogenesis pathways was investigated using quantitative reverse transcription polymerase chain reaction and western blotting. The inhibitory effects of the *L. fermentum* JNU532-derived CFS were mediated by inhibiting the transcription of *TYR, TRP-1, TRP-2*, and *MITF* and the protein expression of TYR, TRP-1, TRP-2, and MITF. Therefore, *L. fermentum* JNU532 may be considered a potentially useful, natural depigmentation agent.

## Introduction

In recent years, the concept of functional food has gradually developed toward the use of dietary supplements that affect the composition and activity of intestinal microbes. Probiotics have been widely incorporated in food and represent live microbe-containing foods, most of which include fermented milk [1]. Lactic acid bacteria (LAB) have been reported to have various beneficial properties and are commonly used as probiotics. Lactic acid bacteria play a crucial role in areas of food fermentation, industrial lactic acid fermentation, and health and medicine.

Recently, genus *Lactobacillus* has been divided into many different genus [2], LAB including *Lactobacillus* species are recognized for their excellent probiotic characteristics. *Lactobacillus* not only enhance the nutritional value and flavor of food but also the functional properties [3]. The growth and survival of LAB depend on conditions in the intestine; certain species can grow in the presence of bile [4]. LAB can improve the digestibility and biological value of food, promote digestion and absorption, reduce cholesterol content in the human body, enhance immune function, and show antitumor and antihypertension activities [5, 6]. In addition, several studies have suggested that probiotics exert certain antioxidant [7], antiageing [8], and skin-whitening [9] effects.

The term “melanin” was first coined by Berzelius in 1840 to refer to black animal pigments [10]. Melanin is a high molecular-weight biological pigment that is usually found in the skin or hair of animals in a polymerized form. Melanin comprises two quinone polymers, namely, eumelanin and pheomelanin. Eumelanin does not contain sulfur atoms and appears brown or black in the skin; pheomelanin contains sulfur atoms and appears yellow or reddish brown in the skin [11]. The description of melanin originates from the study of Bertrand and Bourquelot (1895), who identified tyrosinase in certain mushroom varieties [12]. The complete biosynthesis of melanin was first elucidated by Raper (1920–1930), mainly including melanin cell migration, division, and maturation; formation of melanin bodies; melanin synthesis; operation of melanin particles; and melanin excretion [12]. The process of melanogenesis involves several enzymes, namely, tyrosinase (TYR), tyrosinase-related protein 1 (TRP-1), and tyrosinase-related protein 2 (TRP-2), as well as their transcription factors and diverse signal transduction pathways. The process begins with the metabolism of tyrosine by tyrosinase to produce dopaquinone, which is a key rate-limiting step in the melanin synthesis reaction [13]. Dopaquinone is naturally oxidized to produce dopa and dopa pigment. Dopa also acts as a substrate of tyrosinase, and it can be oxidized to form dopaquinone [14]. Dopa pigment is catalyzed by TRP-2; most of the pigment is decarboxylated to form 5,6-dihydroxyindole (DHI), while a small portion is hydroxylated to form 5,6-dihydroxyindole-2-carboxylic acid (DHICA). DHI is catalyzed by tyrosinase to form indole-5,6-quinone, following which, additional melanin black polymers are produced. DHICA is converted to indole-5,6-quinone carboxylic acid under the action of TRP-1, thereby forming eumelanin; alternatively, DHI may directly produce melanin in a reaction catalyzed by TRP-1. Melanin produced via this pathway is brown; therefore, TRP-1 and TRP-2 play a key role in the synthesis of melanin. Regarding the regulation of melanin synthesis, three of the most common signaling pathways (cyclic adenosine monophosphate, Wnt, and extracellular cell-regulated kinase) involve the action of microphthalmia-associated transcription factor (MITF) [15, 16]. MITF not only participates in the basic physiological activities of melanocytes but is also an important regulatory protein involved in the process of melanin synthesis [16]. MITF can regulate the gene expression of *TYR, TRP-1, and TRP-2*. The upregulation of MITF activates the expression of melanogenesis-related enzymes, thereby stimulating melanogenesis. In contrast, the downregulation of MITF suppresses the expression of melanogenesis-related enzymes, thereby inhibiting melanogenesis [17].

Melanin is a major determinant of skin color and provides defense against the harmful effects of ultraviolet radiation-induced skin damage to a certain extent [18]. Despite its advantages, abnormal melanin production is responsible for the development of several skin diseases, such as albinism, melasma, freckles, and age spots [19, 20]. Therefore, the regulation of melanogenesis is considered an important strategy for treating abnormal skin pigmentation [20]. In recent years, various inhibitors of melanin production, including arbutin, kojic acid, and nicotinamide, have been discovered and used as skin-whitening agents; however, the use of natural inhibitors of melanin production has received more attention than the use of chemically synthesized compounds. This study sought to elucidate the antimelanogenic potential of natural probiotic strains.

Based on the versatility of probiotics, the antioxidation and antimelanogenic activities of probiotic candidates, were investigated in this study. Tested strains isolated from swine intestine, kimchi, and infant feces were screened for their antioxidant properties. Subsequently, the antimelanogenic properties and noncytotoxic activity of selected probiotic candidates were elucidated using B16F10 melanocytes, following which, *L. fermentum* JNU532 was selected. In addition, the mechanism underlying the antimelanogenic activity of *L. fermentum* JNU532 was determined using quantitative reverse transcription-polymerase chain reaction (qRT-PCR) and western blotting.

## Materials and Methods

### Sample Preparation

Twenty probiotic candidates (Table 1) were cultivated in MRS medium (BD, USA) for 24 h at 37°C. The growth rate of 20 tested-strains in 24 h was different. Thus, to unify the number of tested-strains, the absorbance values at 600 nm of the suspensions of the 20 strains were determined and the suspensions were adjusted to the same concentration of 10^8^ cells/ml. Each suspension was centrifuged (3,500 ×*g*, 4°C) for 15 min to obtain a cell-free supernatant (CFS). The CFS was filtered using the 0.2 micrometer syringe (Sartorius AG, Germany) and stored at -20°C.

### Cell Culture

B16F10 cells (ATCC CRL-6475) were maintained in Dulbecco’s modified Eagle’s medium (DMEM)/high-glucose medium (Hyclone, USA) containing 10% fetal bovine serum (FBS) (Gibco, USA) and 1% antibiotic-antimycotic, in an atmosphere containing 5% CO_2_ at 37°C. The medium was replaced thrice in a week. Cells were seeded in 96-well or 6-well flat-bottomed plates. The final volumes were 100 μl/well in 96-well culture plates and 1 ml/well in 6-well culture plates. After culturing for 24 h, the cells were isolated using 0.05% Trypsin-ethylenediaminetetraacetic acid (Gibco) and separated via centrifugation (1,000 ×*g*, 4°C) for 5 min. The supernatant was discarded, and the cell pellets were washed twice with cold phosphate buffer (pH = 7.4).

### Evaluation of Antioxidant Activity

Antioxidant activities of the tested strains were determined using the 2,2-diphenyl-1-picrylhydrazyl (DPPH) scavenging assay, 2,2′-azino-bis (3-ethylbenzthiazoline-6-sulfonic acid) (ABTS) scavenging assay, and ferric reducing antioxidant power (FRAP) assay. The DPPH scavenging activity method developed by Blois (1960) [21] was used, with certain modifications. Briefly, 150 μl of 0.1 mM DPPH (Sigma-Aldrich, USA) solution prepared in methanol and 50 μl of CFS of each strain were added to a 96-well microplate. Control wells contained only methanol, and 0.05 mg/ml ascorbic acid was added as a positive control. The reaction mixtures were incubated at 25°C for 30 min in a dark environment, and the absorbance at 517 nm was measured using a microplate reader (Synergy HTX; Biotek, USA). DPPH scavenging activity was calculated using Eq. (1).

DPPH scavenging activity (%) = [(Absorbance of ascorbic acid – Absorbance of sample)/Absorbance of ascorbic acid] × 100%. (1)

The ABTS scavenging assay used ABTS (Sigma-Aldrich) according to the developer’s instructions. ABTS is oxidized to green ABTS+• by the action of an oxidant, and the production of ABTS+• is inhibited in the presence of antioxidants. An ABTS working solution was prepared in the dark at 12–14 h before performing the assay. The ABTS working solution comprised 7.4 mM ABTS solution and 2.6 mM potassium persulfate, and it was diluted with phosphate-buffered saline (PBS, pH = 7.4). For the ABTS scavenging assay, 150 μl of ABTS working solution and 50 μl of CFS from probiotic candidates were added to a 96-well microplate. The control wells contained only PBS, and 0.05 mg/ml ascorbic acid was added as the positive control. The reaction mixtures were then incubated in the dark at 25°C or 30 min, and the absorbance was measured at 734 nm using a microplate reader (Synergy HTX). The ABTS scavenging activity was calculated using Eq. (2).

ABTS scavenging activity (%) = [(Absorbance of ascorbic acid – Absorbance of sample)/Absorbance of ascorbic acid] × 100%. (2)

The FRAP assay for determining the total antioxidant capacity is based on the principle that under acidic conditions, antioxidants can reduce the blue ferrous tripyridyl triazine (Fe^2+^-TPTZ) produced by ferric tripyridyl triazine (Fe^3+^)-TPTZ. The total antioxidant capacity of the sample can then be determined by measuring the content of Fe^2+^-TPTZ. For the FRAP assay, the working solution of FRAP was freshly prepared by mixing 50 ml of 300 mM acetate buffer (pH = 3.6), 5 ml of 10 mM TPTZ solution (Sigma-Aldrich), and 5 ml of 20 mM iron (III) chloride hexahydrate (FeCl_3_•6H_2_O) (Sigma-Aldrich). The reaction mixture consisted of 150 μl of FRAP working solution and 50 μl of CFS from tested strains in a 96-well microplate; 0.05 mg/ml ascorbic acid was used as a positive control. The reaction mixtures were incubated in the dark at room temperature for 30 min, and the absorbance was measured at 593 nm using a microplate reader (Synergy HTX). A FRAP standard curve was generated using 1 mM iron (II) sulfate hexahydrate (FeSO_4_•7H_2_O), and standard solutions at concentrations of 0, 20, 40, 60, 80, and 100 μM were prepared. The FRAP value was calculated using Eq. (3).

Ascorbic Acid Equivalent FRAP value (%) = (Absorbance of sample/Absorbance of 0.05 mg/mL ascorbic acid) × 100%. (3)

### Cell-Free Tyrosinase assay

L-tyrosine was used as a reaction substrate in the tyrosine activity assay. Briefly, 100 μl of 200 units/ml mushroom tyrosinase (Sigma-Aldrich) prepared in phosphate buffer (pH = 6.5), 50 μl of 1 mM L-tyrosinase, 10 μl of CFS from tested strains, and 40 μl of distilled water (dH_2_O) were added to a 96-well microplate; dH_2_O was used as a negative control, and 500 μM arbutin (Sigma-Aldrich) was used as a positive control. After measuring the initial absorbance at 490 nm using a microplate reader (Synergy HTX), the plate was incubated at 37°C for 30 min, and the absorbance was measured again. The cell-free tyrosinase activity was calculated using Eq. (4).

Inhibition of tyrosinase activity (%) = [(A – B) (C – D)]/ (A – B) × 100%, (4)

where A is the final absorbance of the control, B is the initial absorbance of the control, C is the final absorbance of the sample, and D is the initial absorbance of the sample.

### Cell Viability Assay

Viability of melanoma cells was determined by performing the 3-(4,5-dimethylthiazol-2-yl)-2,5-diphenyltetrazolium bromide (MTT) assay. B16F10 cells (1 × 10^3^ cells/ml) treated with serum-free DMEM containing 10% CFS were incubated at 37°C in humidified air with 5% CO_2_ for 24 h. One hundred microliters of the incubated mixture were replaced with 5 mg/ml of MTT solution (Sigma-Aldrich) dissolved in phosphate buffer (pH = 6.8) and incubated again for 4 h. The MTT solution was removed, and 100 μl of dimethyl sulfoxide (Sigma-Aldrich) was added. Absorbance was measured at 490 nm using a microplate reader (Synergy HTX).

### Measurement of Melanin Content

B16F10 cells (1 × 10^5^ cells/ml) were treated with serum-free DMEM containing 10% CFS. After incubation for 24 h, the medium was removed, and the cells were collected. To measure the melanin content, the cells were dissolved in 1N NaOH at 70°C for 1 h. Cells were grown in DMEM without treatment with CFS as a control. MRS medium (pH = 6.5, pH = 4, pH = 3) was used as a negative control. Absorbance was measured at 490 nm using a microplate reader (Synergy HTX).

### Analysis of Intracellular Tyrosinase Activity

B16F10 cells (1 × 10^5^ cells/ml) were treated with serum-free DMEM containing 10% CFS for 24 h. The B16F10 cells were collected and then lysed using phosphate buffer (pH = 6.8) containing 1% Triton X-100 (Thermo Fisher Scientific, USA) at -80°C for 1 h. After lysis, the cells were centrifuged at 14,000 ×*g* at 4°C for 10 min, and the supernatant was collected. The supernatant (90 μl of cell lysate) and 10 μl of 2 mg/ml L-3,4-dihydroxyphenylalanine (Sigma-Aldrich) were added to 96-well microplates and incubated at 37°C for 30 min. Cells grown in DMEM without CFS were used as a control. Absorbance was measured at 490 nm using a microplate reader (Synergy HTX). Inhibition of tyrosinase activity was calculated using Eq. (5).

Inhibition of tyrosinase activity (%) = (1 – Absorbance of sample/Absorbance of control) ×100% (5)

### Quantitative Reverse-Transcription Polymerase Chain Reaction

B16F10 cells (1 × 10^5^ cells/ml) treated with serum-free DMEM containing 10% CFS were incubated for 24 h. The B16F10 cells were collected, from which the total cellular RNA was extracted using Pure Helix Total RNA Purification Kit (Nano Helix, Korea) as per the manufacturer’s instructions, and the RNA was stored at -70°C. RNA concentration of the samples was diluted to 0.05 mg/μl using dH_2_O. Template RNA (20 μl) was added to Maxime RT-PCR PreMix tubes (iNtRON Biotechnology, Korea). Complementary DNA (cDNA) was synthesized as follows: cDNA synthesis for 60 min at 45°C, reverse transcriptase inactivation step for 5 min at 95°C, dilution of the reactant with sterile water to 50 μl, and storage at -20°C. The cDNA was synthesized using the KAPA SYBR^®^ FAST qPCR Master Mix (2X) Kit (KAPA Biosystems, South Africa), as per the manufacturer’s instructions. The PCR cycles were as follows: denaturation for 30 s at 95°C, annealing for 45 s at 54°C, and extension for 30 s at 72°C. For semi-quantitative assessment of mRNA levels, each PCR reaction was carried out for 35 cycles. A glyceraldehyde-3-phosphate dehydrogenase primer was used as the control. The RT-PCR primer sequences used are listed in Table 2.

### Western Blot Analysis

B16F10 cells (1 × 10^5^ cells/ml) treated with serum-free DMEM containing 10% CFS were incubated for 24 h. This mixture was centrifuged by 3,000 ×*g*, 4°C for 5min and the B16F10 cells were collected. After obtaining the cell pellet, the total protein of the cells was extracted using the PRO-PREP protein extraction solution (iNtRON Biotechnology), and the total protein concentration was determined using the BCA protein assay kit (Thermo Fisher Scientific). The sample protein was mixed with sodium dodecyl sulfate (SDS) sample buffer (200 mM Tris-HCl, 400 mM dithiothreitol, 8% SDS, 6 mM bromophenol blue, 4.3 M glycerol), denatured at 95°C for 5 min, and then placed on ice for 5 min. The sample protein (15 μl) was added to the wells of a polyacrylamide gel and separated using 8% SDS-polyacrylamide gel electrophoresis. Gels were transferred to polyvinylidene difluoride membranes (Bio-Rad, USA) and blocked for 60 min at room temperature in 5% skim milk powder prepared in 0.1% PBST (0.1% Tween 20 in PBS), then washed twice with 0.05% PBST (0.05% Tween 20 in PBS). The membranes were incubated with mouse monoclonal antibodies: β-actin (C4, 1:2000 dilution), TYR (T311, 1:100 dilution), TRP-1 (G-9, 1:100 dilution), TRP-2 (C-9, 1:100 dilution), and MITF (C5, 1:100 dilution) antibodies (diluted with 5% skim milk powder in 0.1% PBST) for 2 h at room temperature or overnight at 4°C. The membranes were washed thrice with 0.05% PBST for 10 min each time, and then incubated with anti-mouse (1:5000 dilution) secondary antibodies (diluted using 5% skim milk powder in 0.1% PBST) for 1 h 30 min at room temperature, before being washed again thrice with 0.05% PBST for 10 min each time. A western blotting detection kit (ELPISBIO, Korea) was used by following the manufacturer’s instructions. Briefly, the membranes were placed in a container, and 0.5 ml of solution A and 0.5 ml of solution B were added to completely cover the membrane before incubation for 1 min. The specific proteins were visualized using the Odyssey Infrared Imaging System (Li-Cor, US). β-actin expression was used as an internal control to demonstrate an equal loading of the protein samples.

### Acid and Bile Acid Tolerance

Tested strains were inoculated in MRS medium (pH = 6.6) at 37°C, and the number of colonies was measured at 0, 24, and 48 h of incubation. The acid tolerance of strains cultured in MRS medium for 18 h was determined, and 100 μl of culture medium was placed in 10 ml of MRS medium containing 1,000 units/ml pepsin (Sigma-Aldrich). The pH was adjusted to 2.5 using HCl at 37°C, and the survival rate of the strains was measured at 0, 1, and 2 h of incubation. Bile salt tolerance was calculated according to the method described by Gilliland *et al*. [3]. Tested strains were cultured in MRS medium for 18 h before 100 μl of culture medium was added to 10 ml of MRS medium containing 0.3% ox gall at 37°C, and the survival rate of the samples was measured at 0, 24, and 48 h of incubation.

### Statistical anAlysis

Results were analyzed using one-way analysis of variance and are presented as the mean ± standard deviation. The analysis was performed using IBM SPSS for Windows Ver. 23 (SPSS, USA), and values of *ρ* < 0.05 (*), *ρ* < 0.01 (**), and *ρ* < 0.001 (***) were considered significant.

## Results

### Antioxidant Activity of the Cell-Free Supernatants of Probiotic Candidates

**Fig. 1:**
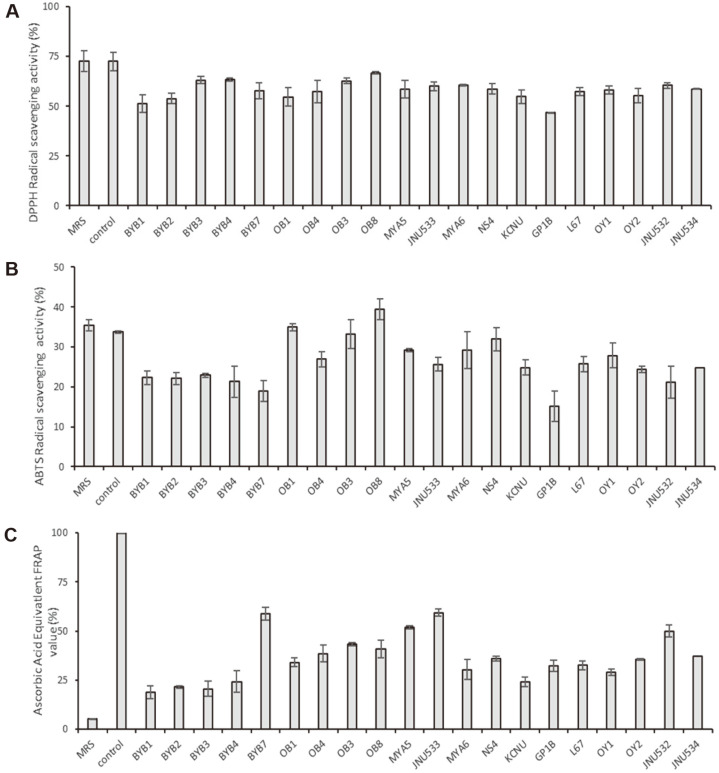
(A) DPPH radical scavenging activity. (B) ABTS radical scavenging activity. (C) Ascorbic acid equivalent FRAP value of cell-free supernatants of tested strains incubated at 37°C for 18 h in MRS medium. Ascorbic acid (0.05 mg/ml) was used as a positive control. The mean values of samples indicated with different letters are significantly different. Data are presented as the mean ± standard deviation (*n* = 3). DPPH, 2,2-diphenyl-1-picrylhydrazyl; ABTS, (2,2′-azino-bis(3-ethylbenzothiazoline-6-sulfonic acid)); FRAP, ferric reducing antioxidant power.

The probiotic candidates identified are listed in Table 1. *Latilactobacillus curvatus* BYB3, *Latilactobacillus curvatus* BYB4, *Levilactobacillus brevis* OB3, *Latilactobacillus sakei* OB8, *Lacticaseibacillus casei* MYA5, *Latilactobacillus sakei* JNU533, *Latilactobacillus sakei* MYA6, *Limosilactobacillus fermentum* NS4, and *Limosilactobacillus fermentum* JNU532 showed approximately 60% DPPH radical scavenging activity. *Levilactobacillus brevis* OB1, *L. brevis* OB3, *L. sakei* OB8, *L. sakei* MYA6, and *L. fermentum* NS4 showed 30%ABTS+• scavenging capacity (Fig. 1). *Latilactobacillus curvatus* BYB7, *L. brevis* OB3, *L. sakei* OB8, *L. casei* MYA5, *L. sakei* JNU533, and *L. fermentum* JNU532 showed > 40% ascorbic acid equivalent FRAP value (Fig. 1). *L. sakei* OB8, *L. casei* MYA5, *L. sakei* JNU533, *L. sakei* MYA6, *L. fermentum* NS4, and *L. fermentum* JNU532 were screened and evaluated via the antimelanogenic activity assay.

### Tyrosinase Activity of the Cell-Free Supernatants of Probiotic Candidates and Intracellular Tyrosinase Activity of Cell-Free Supernatant-Treated B16F10 Cells

**Fig. 2:**
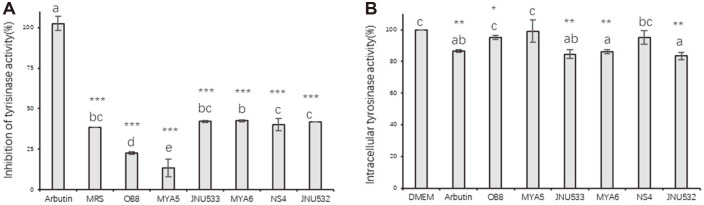
(A) Inhibition of tyrosinase activity by tested strains derived CFS. (B) Tyrosinase activity in B16F10 cells treated with CFS. Arbutin (500 μM) was used as a positive control. The mean values of samples indicated with different letters are significantly different. Significant differences are indicated by different letters according to melanin content from low to high. Experimental groups were normalized to control groups, and the data were analyzed using the t-test. *** *ρ* < 0.001 for 500 μM Arbutin versus sample, respectively. Data are presented as mean ± standard deviation (*n* = 3). CFS, cell-free supernatant.

MRS (as a control) showed 38% tyrosinase-inhibiting activity. *L. sakei* JNU533, *L. sakei* MYA6, *L. fermentum* NS4, and *L. fermentum* JNU532 showed 40% tyrosinase-inhibiting activity; *L. sakei* OB8 and *L. casei* MYA5 showed <30% tyrosinase-inhibiting activity. This suggested that the CFS samples could not inhibit tyrosinase activity to reduce the melanin content. The DMEM control displayed 100% tyrosinase activity, and arbutin inhibited 14% of tyrosinase activity. *L. sakei* OB8, *L. casei* MYA5, and *L. fermentum* NS4 inhibited tyrosinase activity by <5%. *L. sakei* JNU533, *L. sakei* MYA6, and *L. fermentum* JNU532 inhibited tyrosinase activity by >15%(Fig. 2).

### Cytotoxicity and Melanin Content in Cell-Free Supernatant-Treated B16F10 Cells

**Fig. 3:**
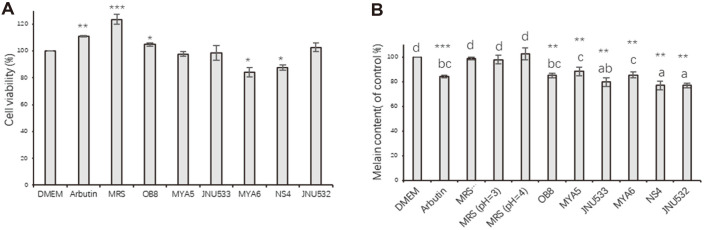
(A) Viability and (B) melanin content of B16F10 cells treated with CFS. DMEM was used as a control. Arbutin (500 μM) was used as a positive control. Significant differences are indicated using different letters according to melanin content from low to high. Experimental groups were normalized to control groups, and the data were analyzed using a t-test. *ρ < 0.05, **ρ < 0.01, ***ρ < 0.001 for control versus sample, respectively. Data are presented as the mean ± standard deviation (*n* = 3). CFS, cell-free supernatant; DMEM, Dulbecco’s modified Eagle’s medium.

DMEM was used as a control. Cells treated with the CFS of *L. sakei* OB8, *L. casei* MYA5, *L. sakei* JNU533, and *L. fermentum* JNU532 showed a viability of 100%. The viabilities of cells treated with the CFS of *L. sakei* MYA5 and *L. fermentum* NS4 were 84% and 88%, respectively. The viabilities of cells treated with 500 μM Arbutin and MRS were 111% and 123%, respectively. The CFS of *L. sakei* OB8, *L. casei* MYA5, and *L. sakei* MYA6 inhibited melanin production by 15%, 11%, and 14%, respectively. The CFS of *L. sakei* JNU533, *L. fermentum* NS4, and *L. fermentum* JNU532 inhibited melanin production by 21%, 23%, and 23%, respectively (Fig. 3). The melanin content in MRS-treated B16F10 cells (pH = 6.5, pH = 4, pH = 3) was the same as that of the control; therefore, it was speculated that the MRS medium (pH = 6.5, pH = 4, pH = 3) had no effect on melanogenesis. Therefore, CFS instead of MRS was chosen for further experiments in the study. Based on the melanin content and intracellular tyrosinase activity, *L. fermentum* JNU532 was selected for further experiments owing to its strong antimelanogenic potential. Subsequently, the mechanisms of the antimelanogenic activity of *L. fermentum* JNU532 were investigated by performing qRT-PCR and western blotting.

### Inhibitory Effect of *L. fermentum* JNU532-Derived Cell-Free Supernatant on the Gene and Protein Expression of *TYR, TRP-1, TRP-2*, and *MITF* in B16F10 Cells

**Fig. 4:**
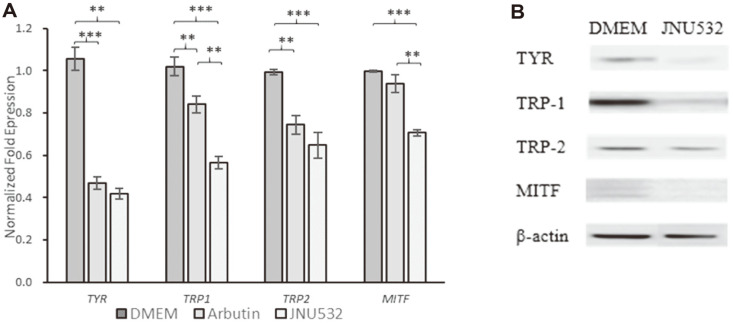
(A) Relative mRNA expression of *TYR, TRP1, TRP2*, and *MITF* in B16F10 cells treated with the CFS of *Limosilactobacillus fermentum* JNU532. DMEM was used as a control. Arbutin (500 μM) was used as a positive control. The mean values of the samples indicated with different letters are significantly different. (B) Inhibitory effects of *L. fermentum* JNU532-derived CFS on protein expression of TYR, TRP1, TRP2, and MITF in B16F10 cells incubated at 37°C for 18 h in MRS medium. DMEM was used as a control. The protein levels of the tyrosinase family of enzymes were analyzed via western blotting, and protein loading amounts were confirmed via β-actin expression. Experimental groups were normalized to control groups, and the data were analyzed using the t-test. **ρ < 0.01, ***ρ < 0.001 for control versus sample, respectively. Data are presented as the mean ± standard deviation (*n* = 3).CFS, cell-free supernatant; DMEM, Dulbecco’s modified Eagle’s medium.

The expression of four genes was reduced in CFS-treated melanocytes (Fig. 4). *L. fermentum* JNU532 CFS may reduce melanin content by inhibiting the expression of key genes required for melanin synthesis including the tyrosinase family genes (especially *TYR*). Western blotting analysis showed that CFS might have inhibited the transcription and expression of melanin-producing genes by suppressing the expression of key proteins TYR, TRP-1, and TRP-2 and blocking the signal of the MITF transduction pathway.

### Acid and Bile Acid Tolerance of *L. fermentum* JNU532

**Fig. 5:**
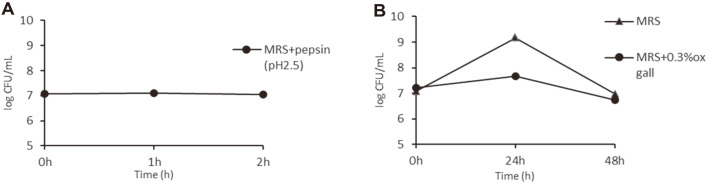
Acid and bile acid tolerances of *Limosilactobacillus fermentum* JNU532.

The results of acid and bile acid tolerance showed that *L. fermentum* JNU532 could survive in the MRS medium (pH = 2.5) for 2 h. Additionally, *L. fermentum* JNU532 showed tolerance to 0.3% ox gall bile salts (Fig. 5).

## Discussion

Melanin is an essential biological pigment. For example, albinism, an autosomal recessive genetic disease, is characterized by the lack of melanin production [11]. Such individuals are very sensitive to sunlight and require protection to avoid damage to the eyes, skin, and other organs caused by ultraviolet radiation [22]. This demonstrates the physiologically protective effects of melanin in humans. However, excessive melanin deposition is physiologically harmful to humans. Several skin diseases are caused by excessive melanin deposition [23]. Therefore, inhibitors of melanin synthesis are widely studied by many researchers.

Recent advances in research on the synthesis of melanin have greatly contributed to the development of melanogenesis inhibitors. The regulation of tyrosinase is crucial for inhibiting melanin synthesis. The mechanism of inhibiting melanogenesis involves inhibiting the activity of tyrosinase [24, 25]. Most of the existing melanogenesis inhibitors directly inhibit the activity of tyrosinase or tyrosine-related proteins [26]. In the contemporary society, demand for skin-whitening products by the young population is increasing. Various foods, medicines, and cosmetics with skin-whitening functions are popular in the current market. Healthy and effective methods for inhibiting melanogenesis are widely applied.

Lactic acid bacteria constitute the most important group of probiotics and are widely used. Although most probiotics have similar effects, LAB from different sources have their own characteristics. Kimchi is a traditional Korean vegetable fermentation product in which a variety of lactic acid bacteria are active during the fermentation process. These bacteria can inhibit harmful intestinal microbiota, lessen the activity of food allergens, reduce mutagenic and carcinogenic activities, display immunomodulatory activity, and lower cholesterol [27]. LAB isolated from the intestine can strengthen the barrier function of the gut microbiota, non-specifically enhance the immune system, maintain the balance of the microbiota, and prevent or remedy gastrointestinal infections [28]. LAB isolated from infant feces reportedly has good probiotic properties [29].

Since different sources have different growth environments, the functions of isolated lactic acid bacteria will also be different. For example, the antioxidant activity may vary from strain to strain. Among the 20 strains of probiotic candidates, 6 displayed the highest antioxidant activities. Some of probiotic candidates with pronounced antioxidant activity inhibit the production of melanin. Among these 6 strains, the CFS of *L. fermentum* JNU532 displayed the highest antioxidant and antimelanogenic activities. This strain could have potential value in the development of natural inhibitors of melanogenesis. qRT-PCR and western blotting show that *L. fermentum* JNU532-derived CFS may downregulate the genes *TYR, TRP-1, TRP-2*, and *MITF* and the proteins TYR, TRP-1, TRP-2, and MITF, which are related to melanogenesis. The results of acid and bile acid tolerance show that *L. fermentum* JNU532-derived CFS can be used as a natural and healthy decolorizing agent in food. A previous study [23] demonstrated that the fermented milk supernatant of *Lactobacillus* inhibited the production of melanin. In our study, however, we studied the CFS of probiotic candidates. In summary, this study demonstrated that *L. fermentum* JNU532-derived CFS exhibited antimelanogenic and antioxidant activities. The findings of this study may aid in the development of skin-whitening products, including cosmetics, food, and medicines.

## Figures and Tables

**Table 1:** Identification of the probiotic candidates tested in this study.

No.	Strains	Abbreviation	Source	Renamed genus [2]
1	*L. curvatus* BYB1	BYB1	Kimchi	*Latilactobacillus curvatus*
2	*L. curvatus* BYB2	BYB2	Kimchi	*Latilactobacillus curvatus*
3	*L. curvatus* BYB3	BYB3	Kimchi	*Latilactobacillus curvatus*
4	*L. curvatus* BYB4	BYB4	Kimchi	*Latilactobacillus curvatus*
5	*L. curvatus* BYB7	BYB7	Kimchi	*Latilactobacillus curvatus*
6	*L. brevis* OB1	OB1	Kimchi	*Levilactobacillus brevis*
7	*L. brevis* OB4	OB4	Kimchi	*Levilactobacillus brevis*
8	*L. brevis* OB3	OB3	Kimchi	*Levilactobacillus brevis*
9	*L. sakei* OB8	OB8	Kimchi	*Latilactobacillus sakei*
10	*L. casei* MYA5	MYA5	Kimchi	*Lacticaseibacillus casei*
11	*L. sakei* JNU533	JNU533	Kimchi	*Latilactobacillus sakei*
12	*L. sakei* MYA6	MYA6	Kimchi	*Latilactobacillus sakei*
13	*L. fermentum* NS4	NS4	Kimchi	*Limosilactobacillus fermentum*
14	*L. amylovorus* CH6	KCNU	Swine intestine	Unchanged
15	*L. acidophilus* GP1B	GP1B	Swine intestine	Unchanged
16	*L. plantarum* L67	L67	Infant feces	*Lactiplantibacillus plantarum*
17	*L. plantarum* OY1	OY1	Kimchi	*Lactiplantibacillus plantarum*
18	*L. plantarum* OY2	OY2	Kimchi	*Lactiplantibacillus plantarum*
19	*L. fermentum* JNU532	JNU532	Kimchi	*Limosilactobacillus fermentum*
20	*L. fermentum* JNU534	JNU534	Kimchi	*Limosilactobacillus fermentum*

*Cultivated in MRS medium for 24 h at 37°C.

**Table 2:** Target genes and sequence of primers used in this study.

Gene	Primer sequences (5’-3’)	References
GADPH	Forward: 5’ –TCACCACCATGGAGAAGGC-3’	Mustachio, L. M, *et al*., (2019)
	Reverse: 5’- GCTAAGCAGTTGGTGGTGCA-3’	
TYR	Forward: 5’ –ACACCTGAGGGACCACTAT-3’	Cai-Jiao Zhang, *et al*. (2017).
	Reverse: 5’- CATTGGCTTCTGGGTAAACT-3’	
TRP-1	Forward: 5’-GCCACAAGGAGGTTAGAAGACA-3’	Cai-Jiao Zhang, *et al*. (2017).
	Reverse: 5’- CCAGTAAGGAAGGGAGAAAGAG-3’	
TRP-2	Forward: 5’- AGAAGTTTGACAGCCCTCC-3’	Cai-Jiao Zhang, *et al*. (2017).
	Reverse: 5’- CAAGTTGCTCTGCGGTTAG-3’	
MITF	Forward: 5'-AACGGGAACAGCAACGAGC-3'	Cai-Jiao Zhang, *et al*. (2017).
	Reverse: 5’-TCACCAGATCAGGCGAGCA-3’	
